# No warning for slow transitions

**DOI:** 10.1098/rsif.2020.0935

**Published:** 2021-03-31

**Authors:** Bregje van der Bolt, Egbert H. van Nes, Marten Scheffer

**Affiliations:** Department of Environmental Sciences, Wageningen University, Wageningen, The Netherlands

**Keywords:** critical transitions, resilience, critical slowing down, dynamic indicators of resilience, regime shifts, rate of forcing

## Abstract

A rise in fragility as a system approaches a tipping point may be sometimes estimated using dynamical indicators of resilience (DIORs) that measure the characteristic slowing down of recovery rates before a tipping point. A change in DIORs could be interpreted as an early warning signal for an upcoming critical transition. However, in order to be able to estimate the DIORs, observational records need to be long enough to capture the response rate of the system. As we show here, the required length of the time series depends on the response rates of the system. For instance, the current rate of anthropogenic climate forcing is fast relative to the response rate of some parts of the climate system. Therefore, we may expect difficulties estimating the resilience from modern time series. So far, there have been no systematic studies of the effects of the response rates of the dynamical systems and the rates of forcing on the detectability trends in the DIORs prior to critical transitions. Here, we quantify the performance of the resilience indicators variance and temporal autocorrelation, in systems with different response rates and for different rates of forcing. Our results show that the rapid rise of anthropogenic forcing to the Earth may make it difficult to detect changes in the resilience of ecosystems and climate elements from time series. These findings suggest that in order to determine with models whether the use of the DIORs is appropriate, we need to use realistic models that incorporate the key processes with the appropriate time constants.

## 1. Introduction

Complex systems can have a tipping point: a threshold point in the conditions after which a self-enforcing feedback brings the system to a new stable state [1–3]. As a result of this feedback, systems with a tipping point have two alternative stable states over a range of conditions. For example, in the tropics, both savannahs and forest-tree cover can be found under a range of mean annual precipitation [4]. In a savannah state, grass fuels fires, thereby maintaining the open landscape. Once the tree cover becomes sufficiently dense, however, the growth of grasses and the resulting fires are suppressed, leading to a self-enforcing shift to a closed canopy state [5–7]. Similar abrupt transitions have been observed in other complex systems, such as shallow lakes [1], coral reefs [1,8] and climate systems [9]. These critical transitions can have long-term dire consequences, because the new state is stabilized by the self-enforcing feedback, making these transitions difficult to reverse [10]. As a result, a wide range of studies try to identify indicators that signal an upcoming transition [11–17]. Most of these indicators are based on the phenomenon that close to the tipping point, the system's resilience to perturbations decreases [18]; close to the tipping point, a system recovers more slowly from a perturbation or disturbance than when it is far from a tipping point. For example, tidal marsh vegetation recovers more slowly from perturbations when inundation stress increases and the system moves closer to a tipping point [19].

A way to quantify the resilience of a system is to estimate the return rate to equilibrium [20,21]. The return rate can be determined using perturbation experiments, but this is often not possible for complex systems such as the climate system. Instead, every system is permanently subject to natural perturbations from the environment. When one monitors the system and its relevant parameters, the system's dynamic responses to these perturbations can be captured and used to estimate the resilience of the system. The resilience is reflected by an increase in the dynamical indicators of resilience (DIORs) variance [22] and temporal autocorrelation [23] of the system state. DIORs have been applied to time series of various complex systems, such as the climate [24], oxygen dynamics in the Mediterranean Sea [25], cyanobacteria populations [26], fresh water lakes [27] and microbial communities [28].

To estimate the DIORs, one needs observational records that are long enough to capture the slowest response time scale of the system [29]. For instance, ‘slow' parts of the climate system, like the deep ocean and ice sheets, respond relatively slowly to changes in the forcing that is on the time scale of humans [29]. A time series of some decades may still be too short to characterize the dynamics of such slow systems.

The sampling frequency—the interval between values in the time series—is also important. Systems need to be sampled at intervals shorter than the characteristic time scales of the slowest return rate [30,31]. At one point, however, it does not help to sample at shorter intervals. For instance, sampling the temperature of the ocean every millisecond does not give more information, because the system does not respond on such short time scales.

These considerations about the length of the time series and the sampling interval are about relative rather than absolute time scales. For instance, the resilience of the postural balance of a person can be determined from a time series with a total length of just 30 s. The typical response rate for physiological meaningful postural control processes is of the order of 3 Hz (0.333 s) [32]. This implies that a time series of 30 s covers many ‘micro-recoveries' of the system. On the other hand, the sampling frequency of 1000 Hz (1 ms) of the equipment is an overkill, as it does not carry meaningful information on the systems response [33].

While such limitations are straightforward, the problem of estimating return times as an indicator of resilience becomes more complicated if the resilience of the system is a moving target, i.e. changing in time. A particularly important example is the situation in which a change in the conditions is slowly moving the system towards a tipping point, where the system shifts to the alternative stable state, such as a lake that is slowly losing resilience due to eutrophication [1]. The typical way to monitor such changing resilience is to calculate the DIORs within a sliding moving window. This method is based on the idea that the DIORs should be estimated as the data are becoming available [12].

A decrease in the resilience of the system could be interpreted as an early warning signal for an upcoming critical transition at the tipping point [34]. This is, however, possible only under a limited set of conditions [35,36]. Otherwise, the probability of false negatives and false positives tends to become very high. In addition, strong environmental perturbations may obscure the trends in the DIORs [37] and can force a system to another state far from the tipping point. For example, when coral reefs shift due to tropical cyclones [38] or when fires cause rapid shifts in vegetation cover [39]. These limitations have led to the suggestion to abandon the term ‘early warning signal' in this context altogether [11].

Nonetheless, there is an obvious demand for early warning signals for critical transitions. For instance, early warning for climate tipping points could have considerable social and economic value for societies [29]. The DIORs have been shown to increase before abrupt climate transitions in the past [24]. However, how abrupt were those transitions really? And how much time would be needed to detect a loss of resilience? The rates of change in the current anthropogenic climate forcing are much faster than in the times for which we studied the ‘early warning signals' for past transitions [40,41]. As the response rates of the oceans and icecaps are rather slow (in contrast to atmospheric systems) we may expect difficulties when it comes to assessing return rates from modern time series.

So far, there have been no systematic studies of the intertwined effects of the slowness of the dynamical system and the rates of ‘forcing' on our chances to detect trends in the resilience from time series. Therefore, we use model-generated data of systems that gradually move towards a tipping point, to quantify how the response rate of the system affects the strength of the resilience indicators prior to a critical transition.

## 2. Methods

### 2.1. Models description

We used four well-studied minimal models with alternative stable states to generate data with different assumptions about the system's response rate (see electronic supplementary material, table S1, for model equations). The first model describes the logistic growth of a resource N that is harvested following a sigmoidal functional response [42]. It describes the transition from an underexploited to an overexploited state as the harvesting pressure crosses a threshold. The second model describes the nutrient dynamics of a lake [43]. At low nutrient input rates, the lake loses nutrients to the sediment or hypolimnion. Once a threshold in the nutrient input rates is passed, there is a high recycling from the sediment or hypolimnion as a result of lower oxygen levels and the lake becomes eutrophic. The third model describes a population with an Allee effect [10,44]. It describes the extinction of a population as the harvest, or loss rate, increases. The fourth model describes the dynamics of tree cover as a function of precipitation [45]. For a range of precipitation levels, this model can be in a high, intermediate or low tree cover state.

In all models, we introduced a parameter, *ɛ*, to control the speed of the system's response. We also assume that each model is subject to random additive independent disturbances, so the general form of each model is

dX=ε ( f(X,c)dt+σdW),

where *f* is the deterministic equation that governs the dynamics of the state variable *X* as a function of *c*, the control parameter which causes the system to switch between stable states. d*W* is a white noise process with a scaling factor *σ*.

### 2.2. Generation of the time series

In order to test whether resilience indicators signal an upcoming transition in slow systems, we ran the model for different values of *ɛ*: ranging from 0.1 to 1 with a step of 0.1. We started from equilibrium and slowly increased the control parameter to make sure that the system crosses the tipping point. We ran the model for an additional 2000 time steps with a constant value of the driver to make sure that the system has enough time to reach the new equilibrium. For each value of *ɛ*, we simulated 100 replicates.

**Figure 1.**
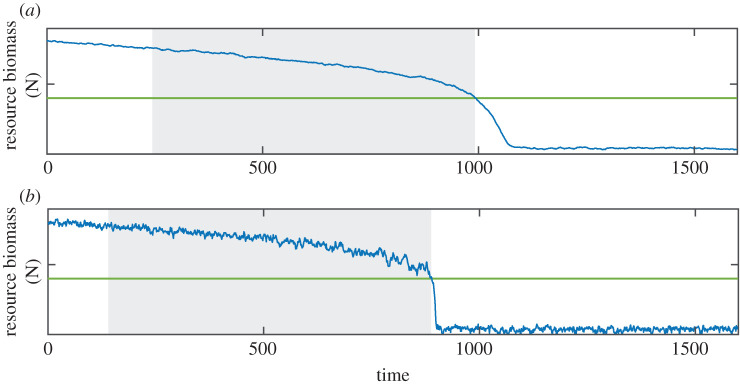
Example time series for a system that responds (*a*) slowly (*ɛ* = 0.1) and (*b*) quickly (*ɛ* = 1). The green lines indicate the threshold value, and the grey area indicates the part of the time series that is selected for standardizing. Time series are generated using the overharvesting model (see electronic supplementary material, table S1).

In the main text, we focus on the results from the overharvesting model, but we analysed the effect of the time scale *ɛ* also for three other minimal models with alternative stable states (see the electronic supplementary material for details). We used only the points in the simulated time series that correspond to the period before the transition. It is important to exclude points that are part of the transition, because due to increased serial correlation and increased variance, including these points would bias the estimate of the resilience indicators [12]. In systems with a low response rate, however, it is difficult to determine the exact onset of the transition, because the decline in the system state is more gradual than in a fast-responding system (figure 1). We defined the transition as the first point where the state variable was smaller than a specific threshold (see electronic supplementary material, table S1) for 10 consecutive time points. The threshold value is based on model runs without external perturbations in a quickly responding system (*ɛ* = 1). In these model runs the system shifts exactly at the tipping point and the moment the transition starts is clearly visible, so one can choose a threshold value for the state variable that is reached before the onset of the transition.

The response rate of the system alters not only the detectability of the resilience indicators but in practice also the length of the time series prior to the regime shift: a lower response rate increases the length of the time series prior to the regime shift, because the shift is delayed. As we focus on the slowness of the system, we standardized the generated time series and selected the 2500 data points prior to the transition. Standardizing the time series removed the statistical effects of differences in time series length of the different response rates of the system. In addition, we compared the performance of the resilience indicators for different levels of time-correlation in the noise process for the overharvesting model (see electronic supplementary material). In these scenarios, the system becomes slow to respond to changes in the forcing and the noise process, because *ɛ* scales both the deterministic equation and the noise process. When only the rate of change of the deterministic part of the model increases, however, this does not necessarily mean that the system also responds more slowly to perturbations. Therefore, we also tested for the overharvesting model how the resilience indicators perform when only the rate of change of the deterministic equations increases (see electronic supplementary material).

To show that a decrease of the response rate of the system is similar to an increase in the rate of change of the driver for discerning a change in DIORs, we performed simulations with the overharvesting model in which we kept *ɛ* constant (*ɛ* = 1), but changed the rate at which we changed the control parameter. In each of these simulations, we increased the value of the control parameter linearly from 1.6 to 2.8 by a fixed rate (0.0024, 0.0012, 0.0006, 0.003) per time step. We include the statistical effect that the time series will likely be shorter when the driver changes rapidly. We take a fixed sampling interval, so if the environmental change is rapid, the system shifts earlier and we have fewer observations prior to the shift (figure 1).

All the simulated time series were produced with the software package GRIND for MATLAB (accessed at http://sparcs-center.org/grind), which used an Euler–Maruyama method to solve the stochastic equation. The estimation of the resilience indicators was performed in R v. 3.4.3 (http://www.r-project.org/) using an adapted version of the R package earlywarnings [12].

### 2.3. Resilience indicators

We calculated two different DIORs: the autocorrelation at the first lag and the standard deviation of the data. To filter out long trends that may cause autocorrelation, we subtracted a Gaussian kernel smoothing function with a predefined bandwidth from the data [12]. The remaining residuals were used for the estimation of the resilience indicators. We estimated the DIORs on the data points within a sliding window of half the size of the time series. We tested for evidence of a trend in the indicators by estimating the nonparametric Kendall rank-correlation tau statistic of the estimates of the DIORs. A strong positive correlation between time and the DIORs indicates a strong trend, which we would expect to occur when approaching a tipping a point [12].

### 2.4. Significance testing

To test whether the trends in the indicators are significant, we calculated the chance that the estimates of the indicators are due to chance alone. We produced the surrogate time series with the same power spectrum and variance as the original time series, but with random phases [46]. For each parameter setting, we generated 100 surrogate time series based on the first replicate of the generated datasets. For each of these surrogate records, we estimated the trend of the resilience indicators (as Kendall's tau) in the same way as the original records. The 97.5th percentiles of the distributions of tau values of each set of surrogate records were considered the lower bound of the confidence interval (*p* = 0.025, single-tailed).

## 3. Results

**Figure 2.**
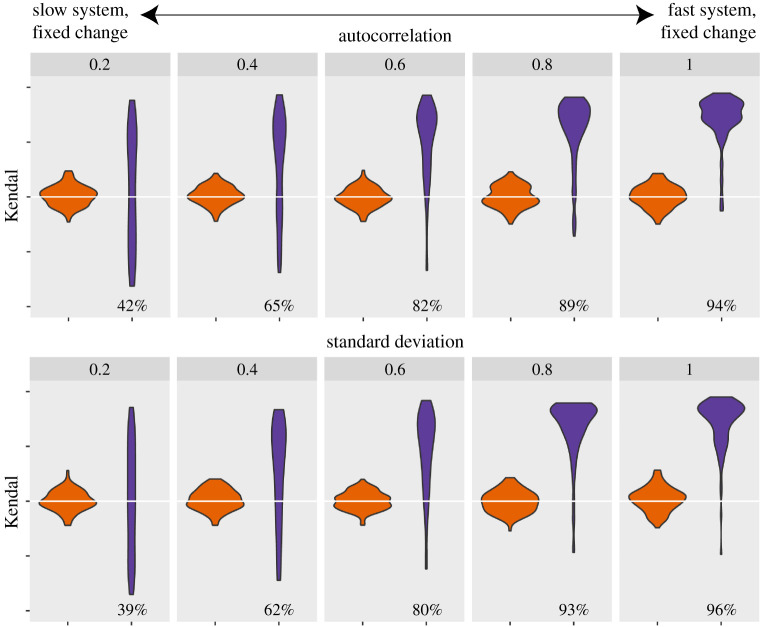
Strength of the trends in autocorrelation for the overharvesting model in the original time series (purple) and null models (orange). The violin plots indicate the distribution of Kendall tau values for the 100 replicates for each level of *ɛ*. The size of the generated datasets is standardized to 2500 points (see Methods). The percentages represent the fraction of trends in the original time series that are significantly higher than the null models (*p* = 0.025, single-tailed).

In all models, we find consistent patterns in the trend of the resilience indicators (DIORs) for the different time scales of the system's response (*ɛ*). Higher *ɛ*'s produce stronger positive trends for both autocorrelation and standard deviation (figure 2 and electronic supplementary material, figures S1 and S2). As the speed of the system's response becomes slower, the number of time series that do not show a significant increase in the resilience indicators increases (electronic supplementary material, table S2). Note that we keep the lengths of the datasets fixed. These results indicate that critical transitions are indeed harder to detect when the response rate of the system is low.

When the noise process is not scaled with the system's response (*ɛ*), the timing of the shift becomes less predictable, but on average the system shifts earlier (electronic supplementary material, figure S3). An earlier transition decreases the predictability of the transition, because the time series before the transition is shorter, and the system shifts further away from the tipping point, where the resilience of the system is higher. When the noise is weaker, the perturbations bring the system less far away from the equilibrium, and the system shifts on average closer to the tipping point, and the variation in the moment if shifting is smaller. In addition, the pattern in the DIORs is similar to the time series in which the noise process is scaled with *ɛ* (electronic supplementary material, figure S4).

When the noise process is time-correlated, the pattern in DIORs is similar for systems with noise processes that are and are not time-correlated (electronic supplementary material, figure S5). When the standard deviation of the noise process is higher, however, the system shifts earlier in a time-correlated environment than in an environment without time-correlation (electronic supplementary material, figure S6), decreasing the predictability of the transition.

**Figure 3.**
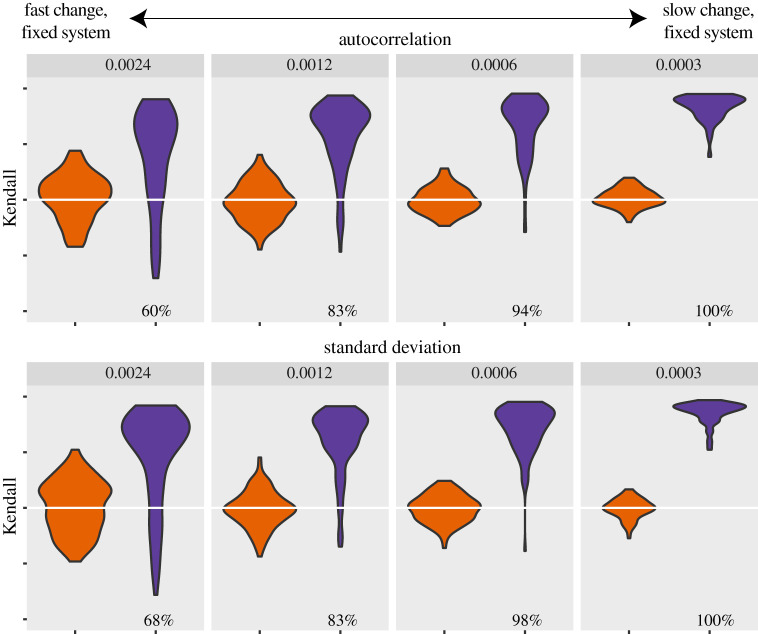
Strength of the trends for the resilience indicators for different rates of change. The violin plots indicate the distribution of Kendall tau values of the indicators determined with the hundred time series (purple) compared to the ones determined with the null model (orange). We fixed the sampling interval so with fast change there are fewer sampling points.

The response rate of the system should be measured relative to the rate of change in the environmental driver. Therefore, we expect a similar effect of an increase in the rate of change in the environment as a decrease in the response rate of the system. To show this, we generated datasets in which the response rate of the system is the same (*ɛ* = 1), but the rate of change of the driver changes. We included here the statistical effect of a faster change on the length of the datasets, assuming that a system is usually sampled at a fixed rate. It is clear that in this scenario the indicators are indeed much harder to detect when the rate of change of the driver increases (figure 3). Also if this statistical effect is excluded by fixing the number of points in the period where the resilience decreases, we still find that DIORs are harder to detect when the rate of the driver is high (electronic supplementary material, figure S7).

## 4. Discussion

The idea that we can use DIORs to detect changes in resilience is based on the assumption that the state of a system is in some kind of equilibrium state [11]. This requires, among other things, that the environmental drivers change slowly compared to the response rate of the system. Clearly, this assumption does not always hold. Some ecosystems respond relatively slowly to changes in the environment [47,48]. Critical slowing down may be difficult to observe in such systems because the response rate of the system is slow to begin with. In this study, we studied the intertwined effects of the response rate of the system and the rate of forcing on our chances to detect critical slowing down from time series. Our analysis shows that the statistical power at slow systems is weaker; therefore, we need more data to detect the resilience indicators in slow systems. Our analysis confirms the suspicion that the rapid rise of anthropogenic forcing to the Earth system may make it difficult to detect changes in the resilience of ecosystems and climate elements from natural time series. In our slowest models, there was still a shift visible. In more extreme cases the tipping point may unfold so slowly that it is hard to distinguish the shift. Such a system may seem to be in equilibrium, but in fact it is unstable and moving on a long transient trajectory to the alternative equilibrium. For instance, fragmented forests in England did not reach their new equilibrium after 1000 years [49]. In ecology, there are systems that have a slow time scale because their key species, like corals and trees, have long lifespans [47], and there are systems that have a delayed response because of an extinction debt that results from habitat destruction [50,51], even if the generation times of the key species are short. For example, plant species diversity in seminatural grasslands in Sweden is significantly related to past habitat connectivity with a time lag of 10–100 years [52], and changes in patterns of certain climatic variables in the early Holocene were so rapid that changes in the climatic limits for certain species exceeded their rates of dispersal and establishment. As a result, some species reached their climatic limits after thousands of years [53,54].

For a system that responds slowly, the period to monitor the system might be too short to detect critical slowing down. Long observational records are needed to determine the slowest response rate of slow systems [29]. For some systems these long records are available. For example, observational records of hundreds to thousands of years have been used to analyse long-term trends in annual growth rates of trees in response to climatic change [55,56]. For other systems, such as the Atlantic Thermohaline Circulation, these records may need to be longer than those that are currently available [29]. Because the climate varies continuously on all time scales, the response rate of the system also depends on the time scale of climatic change on which one focuses [53]. To come back to the example of trees, monthly and annual changes in the climate induce changes in the response rate that can be recorded in tree rings, but to observe decadal to century-scale changes in the climate, long records of pollen data are needed to observe changes in abundance [53]. Since the response rate of a system should be considered relative to the speed of environmental change rather than absolute [53], the effect of the slowness of the system on the DIORs is system and situation specific. In order to assess the specific limitations in more detail, it would be important to use realistic models that incorporate the key processes in any system with the appropriate time constants.

Although the slowness of ecosystems and climate elements may be a problem when it comes to assessing their resilience when limited time is available, there is potentially a bright side to slowness when it comes to our options for managing such change. When the response is slow enough, it may be that even though the theoretical tipping point is already passed, the system responds sufficiently slowly to allow ‘catching it in free fall' and reversing the change [47]. Again, all considerations of time scales are relative. Even extremely rapid quantum jumps in atoms can be caught using real-time monitoring and reversed during their completion [57].

The difference between this example of quantum jumps and the challenge for societies to respond to critical transitions in climate elements or ecosystems is two-fold and contradicting. On the one hand, change unfolds much more slowly, giving us more time. On the other hand, the social response may be slowed down by a complex set of mechanisms [58,59]. For instance, it can be hard to recognize the urgency of a problem if the change is not yet obvious and there are other, more urgent problems on the public agenda. There is also a danger that the change goes so slowly that it is invisible to society because people get used to slowly deteriorating environmental conditions. This so-called shifting baseline syndrome [60] will become especially important if the changes span several human generations. Most importantly, it requires time to reach consensus on action, especially if evidence is weak and urgency is unclear [59]. The latter point is relevant for our line of enquiry as indicators of resilience will be less reliable for slow systems.

## Data accessibility

All data needed to evaluate the conclusions in the paper are present in the paper and/or the electronic supplementary materials. Additional data related to this paper may be requested from the authors.
